# Seasonal Analysis of the Male Reproductive Tract and Germ Cell Proliferation in *Leptodactylus podicipinus* (Anura)

**DOI:** 10.1002/jmor.70072

**Published:** 2025-08-08

**Authors:** Rafael O. A. Bordin, Classius de Oliveira, Raquel F. Domeniconi

**Affiliations:** ^1^ Department of Structural and Functional Biology Universidade Estadual Paulista “Júlio de Mesquita Filho” Botucatu Brazil; ^2^ Department of Biology Universidade Estadual Paulista “Júlio de Mesquita Filho” Sao Jose do Rio Preto Brazil

**Keywords:** PCNA, reproductive anatomy, spermatogenesis, testis

## Abstract

In the reproductive dynamics of anurans, the male gonads have a fundamental relationship with the kidneys. Although reproductive aspects have been widely studied in this group, there are still considerable gaps in understanding the morphology and physiology of the reproductive system of neotropical anurans. Most research has emphasized aspects such as spermatogenesis and reproductive ecology, without information on the structure of the male reproductive tract and the dynamics of spermatogenesis in different species. To better understand the reproductive diversity of anurans, it is essential to comprehend reproductive morphology in a broad sense and simultaneously at different organizational levels. In this context, the present study aimed to characterize the components of the male reproductive tract and assess testicular cell proliferation in *Leptodactylus podicipinus* throughout its reproductive cycle, using histological, immunohistochemical, and computerized microscopy techniques. The male reproductive tract of this species comprises intratesticular ducts that converge into a longitudinal collecting duct, which gives rise to extratesticular efferent vessels entering the kidneys through lateral ducts. These ducts take the sperm through the glomeruli to the collecting ducts, leading to the Wolffian duct. Differences were observed in the intratesticular ducts of individuals in the reproductive and nonreproductive periods. Additionally, the proliferation of the initial germ cells (spermatogonia and spermatocyte I) exhibited positive PCNA staining, with distinct differences between the two periods analyzed.

## Graphical abstract

Box 1: Anuran reproductive morphology and dynamics are complex and diverse. Box 2: It is essential to comprehend reproductive morphology in a broad sense and simultaneously at different organizational levels. Box 3: The study characterizes the components of the male reproductive tract and assess testicular cell proliferation throughout the reproductive cycle of *Leptodactylus Podicipinus*. Box 4: The male reproductive tract of the species present an intratesticular longitudinal collecting duct and seasonal differences in the morphology and testicular cell proliferation.

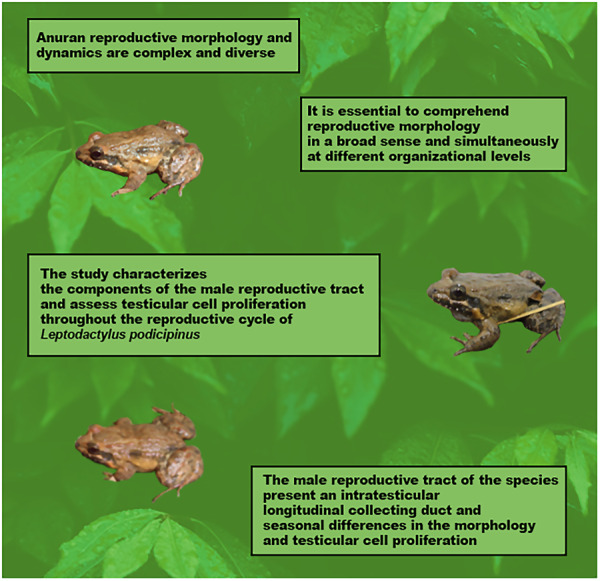

## 1 Introduction

The reproductive tract of vertebrates has been widely studied due to the anatomical and functional variability between different zoological groups (Lombardi 1998). From an evolutionary perspective, the origin and development of the reproductive tract in vertebrates occur in close association with the urinary tract, rendering them practically inseparable in many groups. This relationship can be explained by the fact that both originate from adjacent mesodermal structures during embryonic development (Oielska 2009). From these mesodermal elements originates the most ancestral nephric portion, i.e., the pronephros, which develops into a structure with glomeruli, i.e., the dorsal kidneys or opistonephros, present in anamniotes (cyclostomes, aquatic gnathostomes [‘fish’] and amphibians (Lombardi 1998). The male reproductive tract is characterized by the production of sperm in the testes and the transport of these cells through a network of canals located inside and outside the gonads. These channels run through the testes and kidneys and end in the cloaca (Oielska 2009; Rheubert et al. 2017). The interaction between the kidneys, testes, and genital processes is a common feature in many anuran species. For example, the cranial part of the kidneys, known as the reproductive kidney, plays a crucial role in sperm transport. (Oielska 2009; Hiragond and Saidapur 2000; Rheubert et al. 2017).

However, this association can vary between species, including patterns in which the kidneys do not participate in gamete transport (Blüm 1986; Oielska 2009). The connection between the urinary and genital portions, especially in males, occurs during sexual differentiation (Lombardi 1998). In amphibians specifically, this connection also occurs through the connection of the primordial seminiferous parenchyma with the ducts called *vasa efferentia* that run from the testes through the mesorchium or mesothelium towards the kidneys (Oielska 2009; Rheubert et al. 2017; Serrano‐Perez and Ramírez‐Pinilla 2021).

Among the three orders of amphibians, Gymnophiona, Caudata, and Anura, the morphological patterns of the testes and the sperm duct show both particularities and similarities. In apodes (Gymnophiona), the lobed testes are connected to the kidneys and the Wolffian duct by several transverse efferent vessels, which originate from a central canal in the testes (Gomes et al. 2012; Serrano‐Pérez and Ramírez‐Pinilla 2021). In urodeles (Caudata), the testes are organized internally into seminiferous tubules that connect to a central canal, similar to the apodes, which branch into several efferent vessels, that run from the testes towards the kidneys. These vessels transport the sperm to the renal corpuscles or directly to the proximal renal ducts, following through the renal canals to the Wolffian duct (Siegel et al. 2014).

Although there are several studies on the male reproductive tract of amphibians, especially anurans, it is important to note that the morphology and histology of these structures vary widely between different species (Hiragond and Saidapur 2000; Rheubert et al. 2017). Despite a large amount of research on anuran reproduction, many focus on ecological, evolutionary, or cellular aspects (Santos et al. 2011; Pucci Alcaide et al. 2020; Bordin et al. 2024b), leaving gaps in knowledge about the detailed anatomical description of the reproductive tract of various species (Rheubert et al. 2017).

The morphology of reproduction in anurans is a complex and promising field with great potential for discovery. The male reproductive process involves a number of factors, including the development of the gonads, spermatogenesis, the maintenance of spermatozoa, and their transportation to ensure reproductive success (Méndez‐Tepepa et al. 2023). Several studies have investigated the dynamics of spermatogenesis in different anuran species (Santos et al. 2011; Chaves et al. 2017; Pucci Alcaide et al. 2020; Bordin et al. 2022). In this context, cell proliferation in the gonads is a fundamental aspect of understanding testicular dynamics in anurans, especially concerning the type of cell involved in spermatogenesis (Chieffi et al. 2000).

The use of proliferating cell nuclear antigen (PCNA) has been effective in analyzing cell proliferation in anuran testes, and this tool has been related to the species' seasonal reproductive cycles (Chieffi et al. 2000; Chianese et al. 2015; Olea et al. 2021). Understanding cell biology is one of the essential aspects for comprehending the integral functioning of reproduction. Furthermore, knowledge of cellular processes is fundamental to understanding the biological patterns observed in the reproductive cycles of species.

Considering the importance of anatomical and cellular information in the study of the reproductive system and the scarcity of data relating these aspects to reproduction in the anuran group, this study aims to describe the structure of the male reproductive tract of *Leptodactylus podicipinus* (Leptodactylidae). It will be compared with available data on other amphibian species, and cell proliferation in the testes of this species will be analyzed using PCNA immunolocalization throughout its reproductive cycle. We hypothesize that the anatomical and cellular aspects of the individuals of this species vary seasonally.

## 2 Methods

### 2.1 Study Species

*L. podicipinus* (Cope 1862) is widely distributed in the Neotropical region, being found in Brazil, Bolivia, and Paraguay, northeastern Argentina (Sá et al. 2014). In terms of reproductive behavior, the species is commonly found in open areas on the edges of ponds or pools with undergrowth, where it breeds in natural or constructed shallow basins (de Almeida Prado et al. 2000; Bordin et al. 2024a).

### 2.2 Sampling

Collections were carried out over 2 years (2021–2023). Individuals were located and captured in the field using active search methods and listening at vocalization sites. Field trips were made from dusk until late at night (6 pm to 11 pm). Sexual maturity was confirmed through secondary sexual characteristics such as the presence of vocal sacs and spines on the hands. The individuals were separated into two groups according to the different periods of the species' reproductive cycle (Bordin et al. 2024a), a reproductive period from October to February (REP—11 individuals) and a nonreproductive period from March to September (NREP—9 individuals).

The sites where the individuals were obtained were Porto Said (22°40′28.0“S 48°20′00.8” W) and Rio Bonito (22°40′49.1“S 48°19′37.0” W), both located in the municipality of Botucatu—São Paulo, Brazil. The locations have an average annual temperature of 21°C and annual rainfall of 1500 mm (Franco et al. 2023). The specimens were euthanized through the topical application of 5% lidocaine to both the dorsal and ventral surfaces.

The animals used in this experiment were kept under the Ethical Principles in Animal Experimentation, adopted by the Brazilian College of Animal Experimentation (CEUA No. 4478221021), and all collections were carried out under a license to collect and keep amphibians in the laboratory (No. 79953‐3 SISBIO/IBAMA).

### 2.3 Scanning Electron Microscopy

To obtain scanning microscopy images, an independent collection of 3 adult males from the reproductive period was made. The testes were collected and fixed in buffered glutaraldehyde (2.5%) at room temperature for 3 h. They were then dried to the critical point using acetone at a temperature of 4°C. After the drying process, the samples were sputter‐coated with gold and taken to the scanning electron microscope (Quanta Series 200—FEI Company), using an accelerating voltage of 12.5 KeV. The technique was performed at the Electron Microscopy Center of the Universidade Estadual Paulista, Botucatu campus.

### 2.4 Histology

Histological analyses were carried out to evaluate the architecture of the reproductive tract of *L. podicipinus*. Twenty sexually mature adult males were used (REP—11; NREP—9). The testes and kidneys were extracted and fixed in buffered formalin (10%) and embedded in Paraplast (Sigma–Aldrich) following standard protocols (Canene‐Adams 2013). Non‐serial 3 µm‐thick sections of the testes and kidneys were obtained using a rotary microtome (longitudinal sections). The material was stained with Hematoxylin‐Eosin (HE) and analyzed using light microscopy (Axiophot 2 microscope with AxioCam HR, Zeiss, Germany) at the Anatomy Laboratory of the Instituto de Biociências de Botucatu—Unesp.

The sections from individuals from both reproductive periods were compared qualitatively and classified into two categories based on the morphology of the intratesticular ductules: (1) dilated with sperm, and (2) closed or empty. This classification was performed to identify morphological changes in the testes throughout the reproductive cycle of the species.

### 2.5 Immunohistochemistry

Histological sections of the testes were deparaffinized in xylene and hydrated in ethanol. Antigen retrieval was carried out by incubating the sections in citrate buffer (pH: 6.0) at 100°C for 15 min in a microwave oven (power 750 W). Endogenous peroxidases were blocked in methanol (3% H2O2) for 15 min. To reduce nonspecific protein‐protein bonds, the sections were incubated in a blocking solution with bovine serum albumin (3%) in PBS‐T buffer for 1 h at room temperature. Subsequently, the Anti‐PCNA antigen was localized using the primary antibody diluted at a concentration of 1:100 (MAB424—MiliporeUSA) in 1% BSA and incubated overnight at 4°C. After washing with PBS‐T buffer, the sections were incubated with the HRP secondary antibody (1:100 anti‐mouse Abcam) for 1 h at room temperature and then revealed with diaminobenzidine (DAB) and counterstained with Mayer′s hematoxylin. Sections with the primary antibody replaced by 0.01 mol l^−1^ PBS were used as negative controls.

The sections were dehydrated and coverslipped for imaging and analysis using an Axiophot 2 photomicroscope equipped with an AxioCam HR digital camera (Zeiss, Jena, Germany), from the Department of Structural and Functional Biology, Anatomy Sector, Instituto de Biociências de Botucatu, UNESP‐Universidade Estadual Paulista, Brazil.

### 2.6 Proliferating Cell Nuclear Antigen Quantification

The images obtained from the slides using the immunohistochemistry technique were analyzed using Image ProPlus software (v6.0, Media Cybernetics). The cells marked by the PCNA antibody were quantified using Weibel's graticulated method (Weibel 1963). For this, the values were obtained by counting points, and a total of 50 images were analyzed, approximately 3 to 6 images per individual analyzed.

### 2.7 Comparison With Other Species

To establish a framework for comparative anatomy, data were compiled from existing literature on amphibian species with detailed descriptions of their reproductive systems. The anatomical features compared included internal testicular morphology, the presence of a central intratesticular duct, and the structure of the extratesticular ducts. The species included in the comparison were *Aquarana catesbeiana* (Ranidae, Anura), *Caecilia thompsoni* (Caeciliidae, Gymnophiona), and *Ambystoma maculatum* (Ambystomatidae, Caudata).

### 2.8 Statistical Analysis

The Mann‐Whitney non‐parametric test was used to compare the means of the groups. Before applying the test, the data were analyzed for normality using the Shapiro–Wilk test. The statistical analyses were carried out using the R software (version 4.3.2).

## 3 Results

### 3.1 Male Reproductive Tract

**Figure 1:**
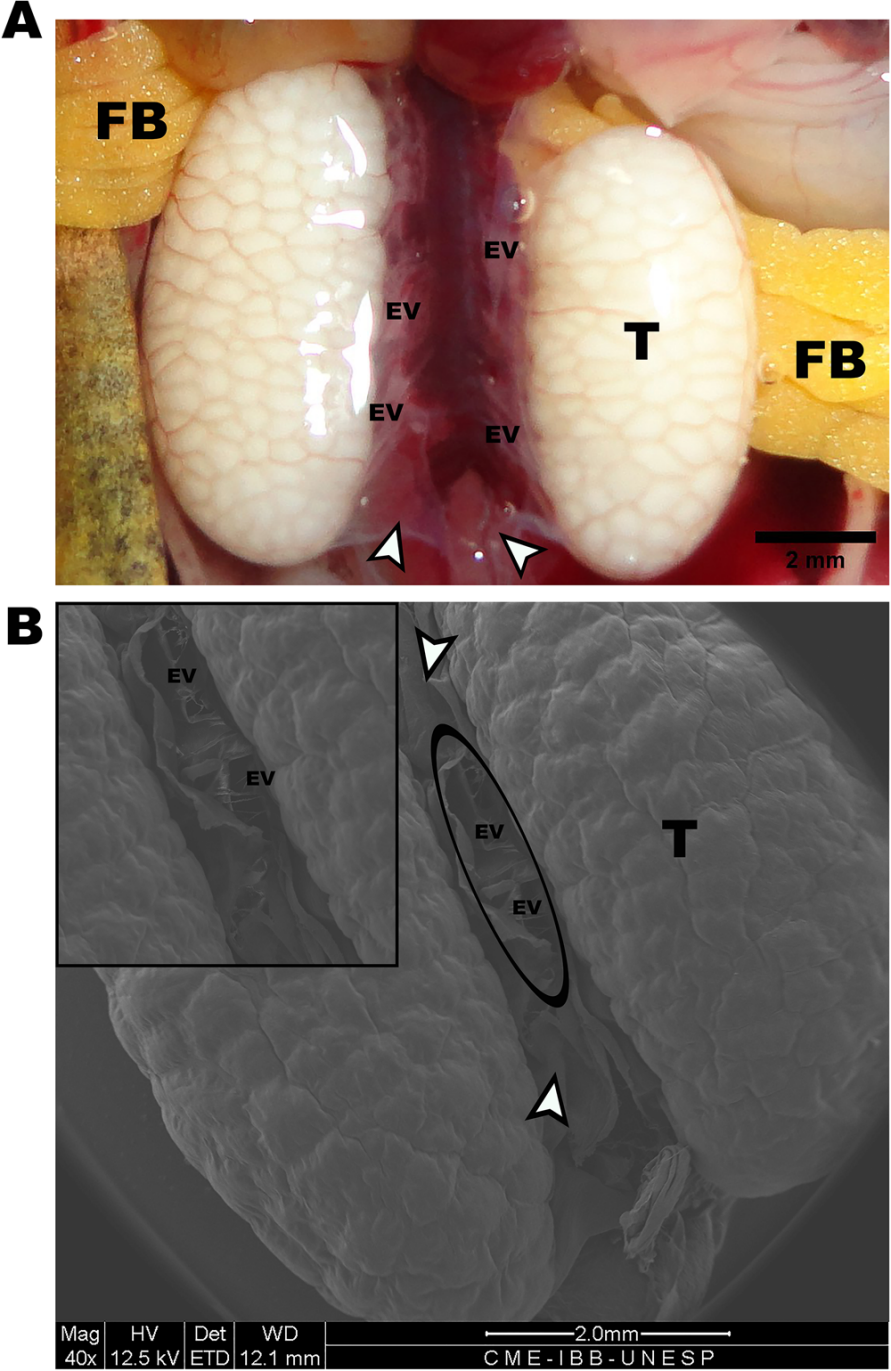
*Leptodactylus podicipinus*, anatomy of the reproductive tract. (A) Macro‐photograph of efferent vessels leaving the testis in the direction of the dorsal kidneys; the testis is surrounded by the mesorchium (arrowheads); cranial fat bodies can be observed in the cranial part of the testes. (B) Scanning electron micrograph of the reproductive tract; testicular surface; the efferent vessels exposed after cutting the mesorchium. Arrowheads – mesorchium; EV – efferent vessels; T – testicular surface; FB – fat bodies.

The reproductive tract of males of the species *L. podicipinus* consists of two whitish ovoid testes located in the abdominal cavity (Figure 1). The kidneys are located dorsally to the testes, and both the kidneys and the ducts and blood vessels observed are supported by a visible fibrous tunic, i.e., the mesorchium, which also surrounds the testes (Figure 1). The testes within the mesorchium, in turn, are covered by another fibrous tunic, the tunica albuginea, which, due to its transparency, makes it possible to identify the peripheral testicular architecture by visualizing the seminiferous loculi (parenchyma) being delimited by the arrangement of the stroma.

Internally, the germinative epithelium develops within the sac‐like parenchyma, that is, the seminiferous loculi. Within the same locule, there are different clusters of germ cells at varying stages of maturation, these clusters are germ cysts and are supported by Sertoli cells from the periphery of the loculi. Between the loculi of the parenchyma are somatic support cells that form the stroma, including fibroblasts that delimit the loculi and Leydig cells.

**Figure 2:**
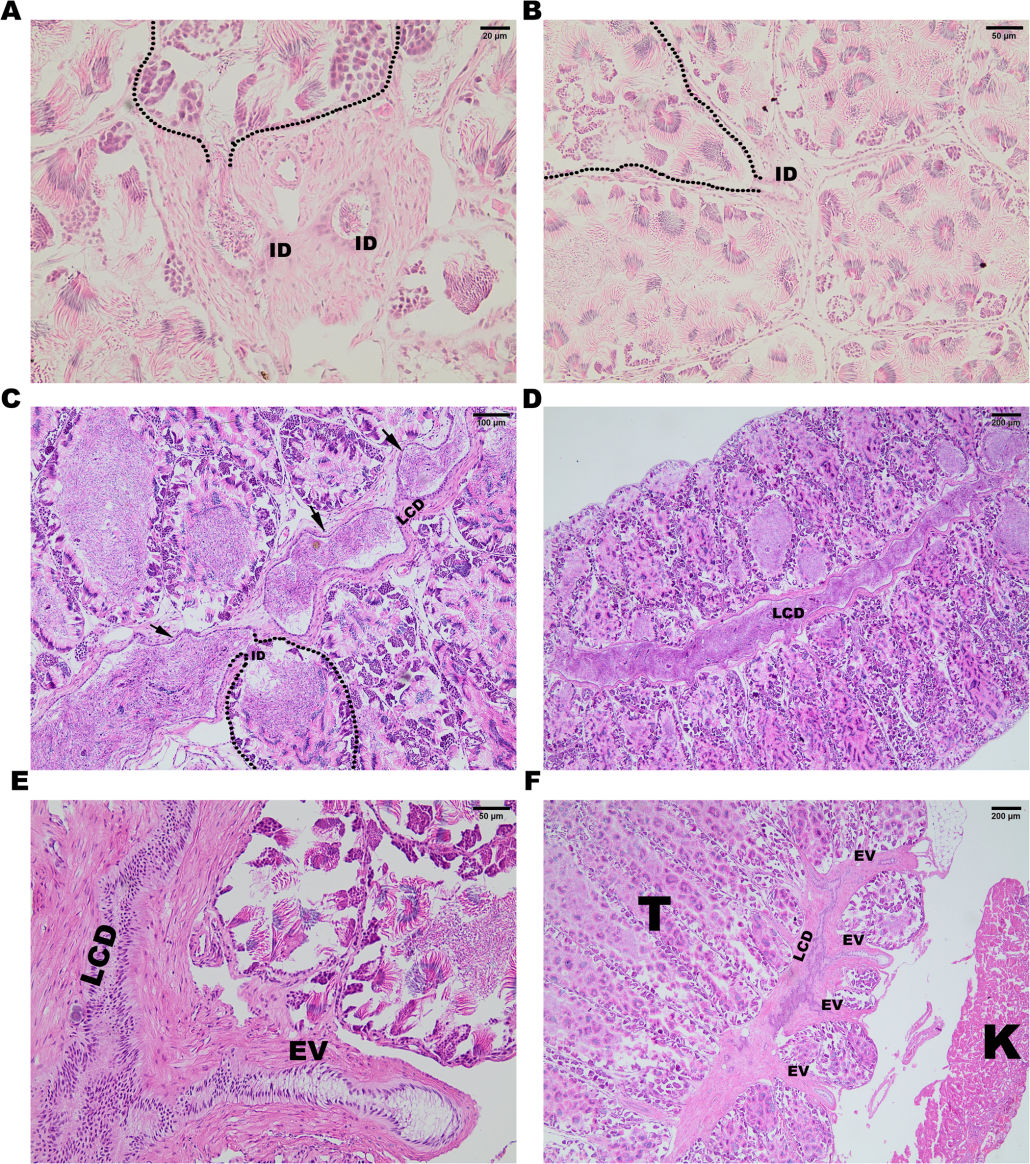
*Leptodactylus podicipinus*, photomicrograph of the histological details of the reproductive tract. (A and B) Intratesticular ductules drain the sperm from the seminiferous locules. (C and D) The draining intratesticular ductules fuse, creating larger ductules that later fuse in the longitudinal collecting duct. (E and F) Efferent vessels, which branch from the longitudinal collecting duct and cross the mesorchium towards the kidney. Dotted lines – Loculi delimitation; ID – intratesticular ducts; Arrows – Large IDs before fusion; LCD – longitudinal collecting duct; EV – efferent vessels; K ‐ renal corpuscle and T ‐ testis. A bar: 20 µm/B and E bar: 50 µm/D bar: 200 µm/F bar: 200 µm/C and F bar: 50 µm. Dotted line—delimitation of the locules. Hematoxylin and Eosin (HE) staining.

After spermatogenesis, mature free sperm are found in the locular lumen. Intratesticular ductules (ID) can be seen draining the seminiferous loculi (Figure 2A,B). These ductules converge into a larger common channel, called the longitudinal collecting duct (LCD—Figure 2C,D; Figure 4). The sperm are then transported to the extratesticular ducts, i.e., efferent vessels (EV —Figure 2E,F; Figure 4), which cross the mesorchium and can be seen between the testes (Figure 1A).

**Figure 3:**
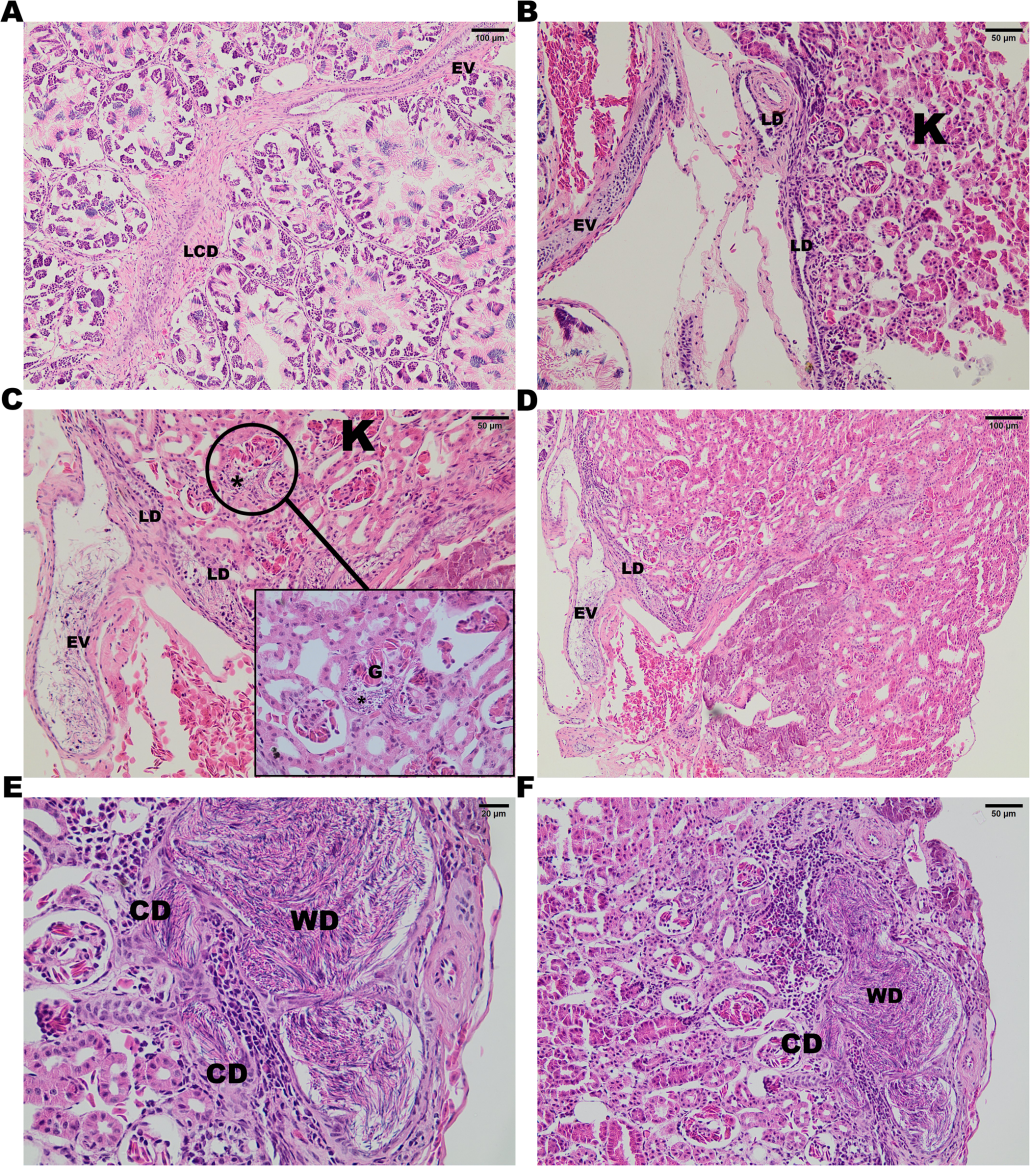
*Leptodactylus podicipinus*, photomicrograph of the histological details of the reproductive tract in *L. podicipinus*. (A and B) The longitudinal collecting duct branches in efferent vessels towards the kidney, where it connects to the lateral ductules of the kidney. (C and D) The lateral ductules carry the sperm through the glomerulus. (E and F) The renal collecting duct transports the sperm to the Wolffian Duct. LCD – longitudinal collecting duct; EV – efferent vessel; LD – lateral ductule; K – Kidney; G – glomerulus; * ‐ sperm; CD – renal collecting duct and WD – Wolffian duct. A and D bar: 100 µm/B, C and F bar: 50 µm/E bar: 20 µm. Hematoxylin and Eosin (HE) staining.

**Figure 4:**
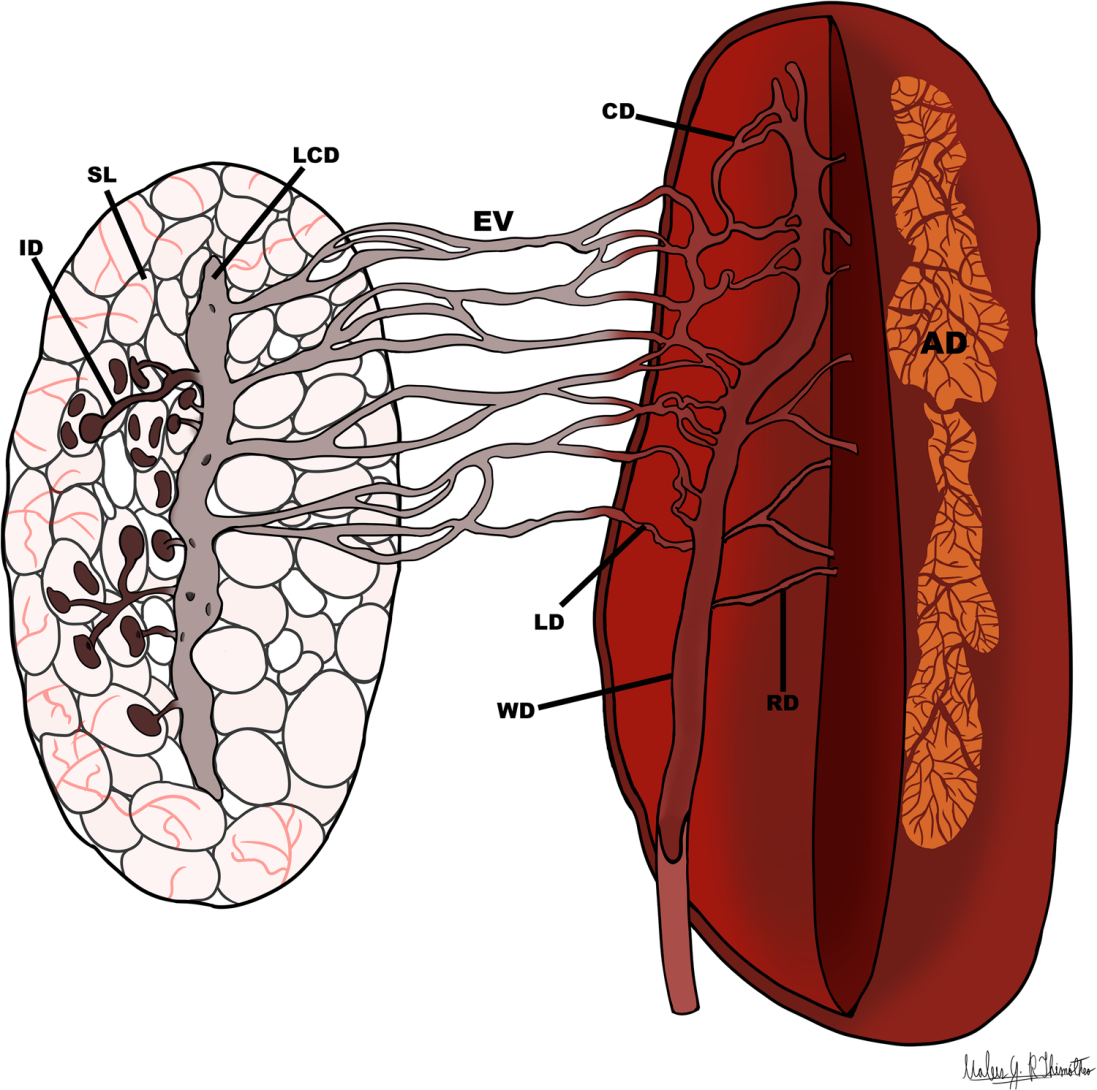
*Leptodactylus podicipinus*, anatomical drawing of the reproductive tract. The illustration depicts the sperm transport pathway, beginning in the seminiferous locule. Sperm is first drained by the intratesticular ductules, which convey it to the longitudinal collecting duct. From there, sperm pass into the efferent ducts that traverse the mesorchium and enter the kidney, ultimately reaching the lateral ductules of the kidney. These ducts channel the sperm through the glomeruli and into the collecting ducts, which lead to the Wolffian duct. SL – Seminiferous locule; ID – intratesticular ductules; LCD – longitudinal collecting duct; EV – efferent vessels; LD – lateral ductules; CD – collecting ducts; RD – renal ducts; WD – Wolffian duct; AD – Adrenal gland.

The efferent vessels (EV) then connect to the lateral ductules of the kidneys (LD—Figure 3A,B) and finally enter the reproductive part of the kidneys (Figure 3C–F; Figure 4), where the sperm are transported through the network of lateral ductules to the renal collecting ducts (CD), which take the sperm to the Wolffian duct (WD—Figure 3E,F; Figure 4).

**Figure 5:**
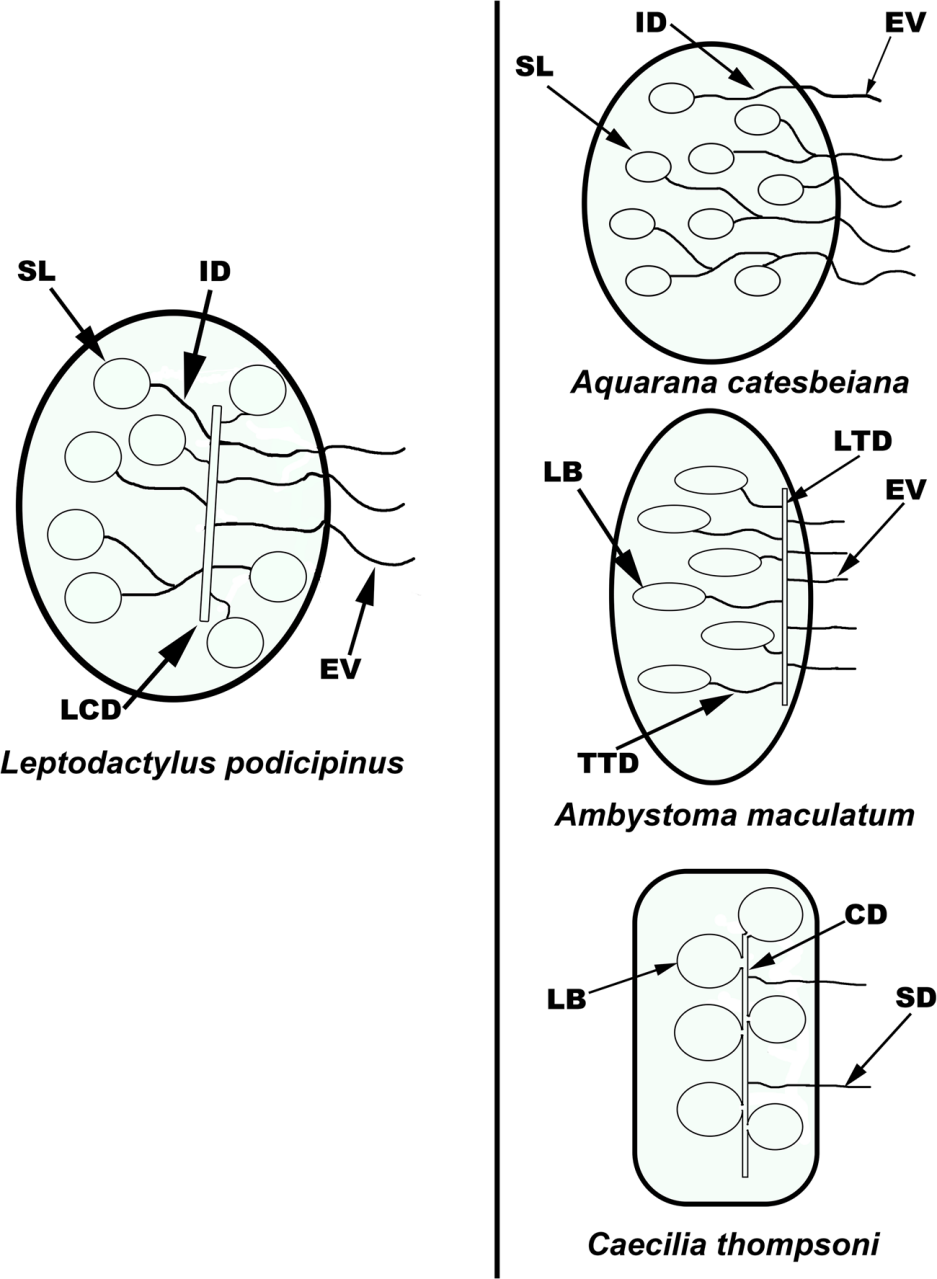
Comparative diagram of the reproductive tract of amphibian species. Schematic representing the male reproductive tract of *L. podicipinus*, *A. catesbeiana*, *Ambystoma maculatum*, and *C. thompsoni*. SL ‐ seminiferous loculus; ID ‐ intratesticular ductules; LCD ‐ longitudinal collecting duct; EV ‐ efferent vessels; LB ‐ seminiferous lobule; TTD ‐ transverse testicular duct; LTD ‐ longitudinal testicular duct; CD ‐ spermatic collecting duct; SD ‐ spermatic duct. **Schemes based on and adapted from** Wake 1968; Siegel et al. 2013; Rheubert et al. 2017; Serrano‐Perez and Ramírez‐Pinilla 2021.

By comparing the male reproductive tract of *L. podicipinus* with data from the literature on other amphibian species, including representatives of the orders Gymnophiona and Caudata, a shared morphological feature was identified between *L. podicipinus* and the apode and urodele species (Figure 5). Specifically, the longitudinal collecting duct (LCD—*L. podicipinus*), longitudinal testicular duct (LTD—*Ambystoma maculatum*) and spermatic collecting duct (CD—*C. thompsoni*) is a similar morphological aspect shared by the three species. *A. catesbeiana* and *L. podicipinus* present a similar organization of seminiferous loculi (SL) and intratesticular ducts (ID), in contrast with the seminiferous lobules (LB) in *Ambystoma maculatum*; however, *A. catesbeiana* does not present a longitudinal duct. A comparable structure found in all three species is the branching extratesticular ducts towards the kidneys (EV and SD in *C. thompsoni*, Figure 5).

**Figure 6:**
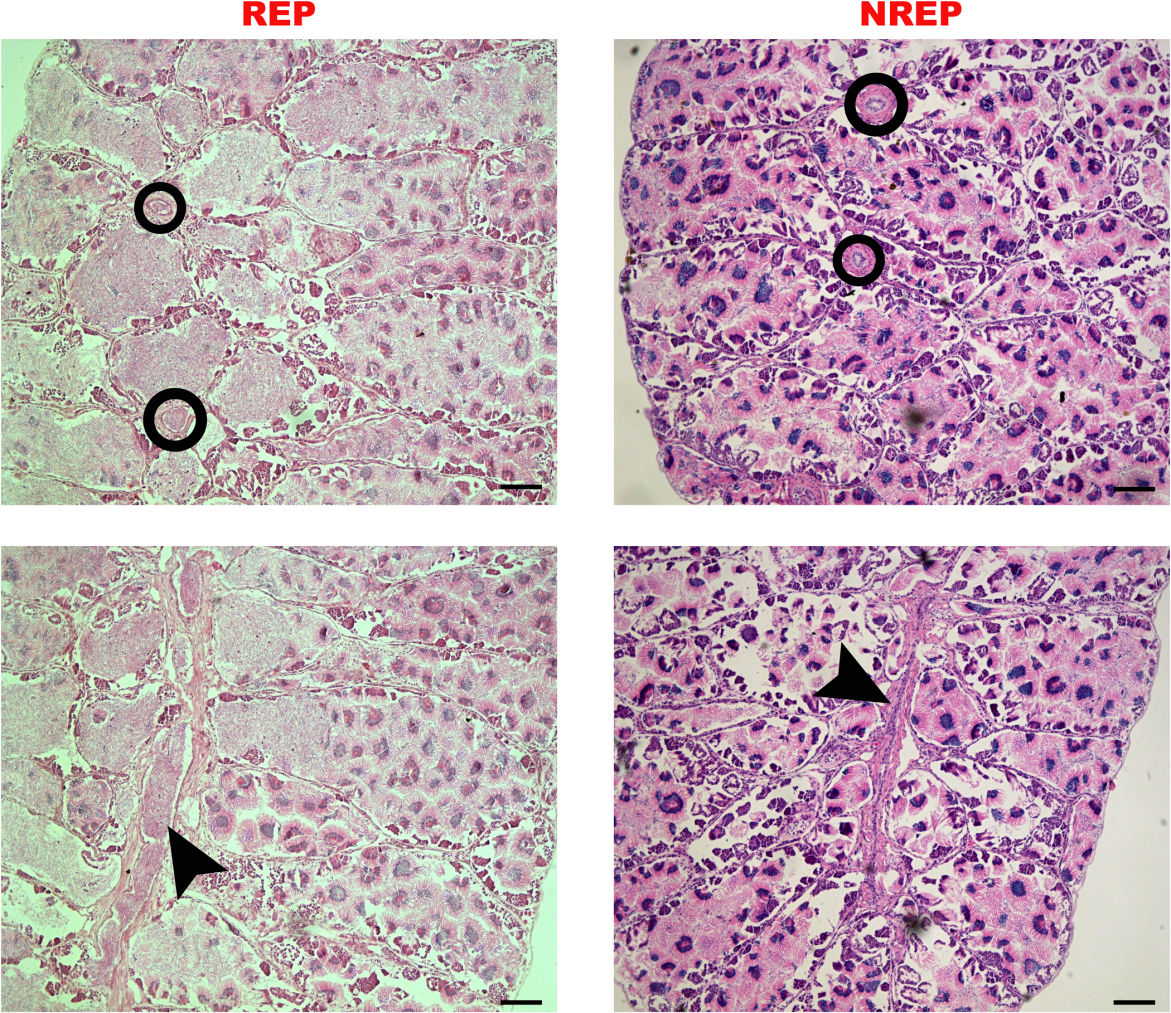
*Leptodactylus podicipinus*, comparison of the histological characteristics of the intratesticular ducts in different reproductive periods. Bar: 100 µm. Circles ‐ Intratesticular ducts; Arrowheads ‐ Longitudinal collecting ducts. REP ‐ reproductive, NREP ‐ nonreproductive.

Concerning the periods during the reproductive cycle (REP and NREP) of the species, it was possible to observe differences in the intratesticular ductules of the individuals studied. Most of the individuals from the nonreproductive period (Table 1) had closed ducts and ductules without many spermatozoa (Figure 6). In contrast, individuals from the reproductive period had the ductules (Table 1) dilated and filled with sperm varying in quantity (Figure 6). In general, the pattern of the animals from the reproductive period was of dilated intratesticular ductules and longitudinal canal with sperm, while the animals from the nonreproductive period had a pattern of ductules and longitudinal canal closed, empty, or with few sperm.

**Table 1:** Proportion of testicular ducts observed in *L. podicipinus* in the different periods studied.

Period (*n*=20 individuals)	Testicular ductules	
	Dilated with sperm	Closed or empty
REP (*n *= 11)	63.6%	36.4%
NREP (*n* = 9)	22.3%	77.7%

Abbreviations: NREP, nonreproductive; REP, reproductive.

### 3.2 Proliferating Cell Nuclear Antigen Analysis

**Figure 7:**
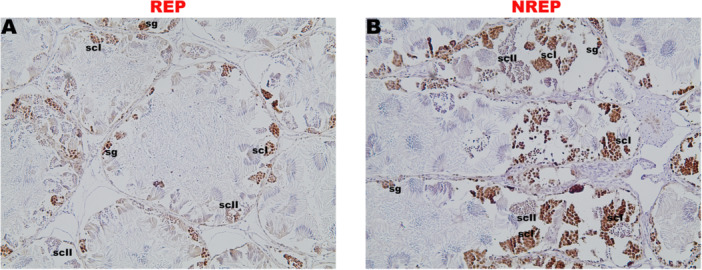
*Leptodactyluspodicipinus*, PCNA immunolocalization in the testes. Animals from different periods were used REP ‐ reproductive and NREP ‐ nonreproductive. sg ‐ spermatogonia; scI ‐ primary spermatocyte; scII ‐ secondary spermatocyte.

In the analysis of PCNA immunoreactivity in the testes of *L. podicipinus* individuals, the germ cells of earlier stages were positively marked, such as spermatogonia and spermatocytes (Figure 7), while later cells such as spermatids and spermatozoa showed no prominent immunoreactivity. Positive labeling was observed in both the nucleus of the cells and the cytoplasm.

**Figure 8:**
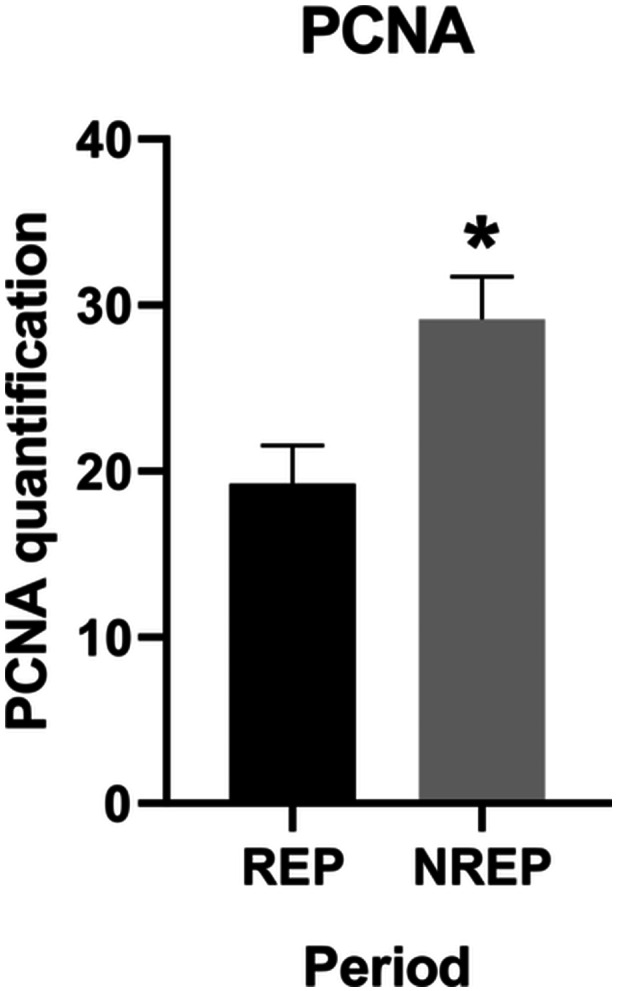
*Leptodactylus podicipinus*, graph of PCNA quantification of individuals from the periods tested. Mann‐Whitney test; significance *p* < 0.05; values expressed as the mean and standard error of the mean. REP – reproductive period, NREP – nonreproductive period.

Immunohistochemistry was carried out on the testes of animals from different periods of the species' reproductive cycle. When the amount of PCNA was compared between individuals from the two periods studied, there was a significant difference (Mann–Whitney *U* = 17, *P* < 0.05 two‐tailed) between the periods. Individuals of *L. podicipinus* from the nonreproductive period (NREP) had a higher average number of positive PCNA markings in the testis than animals from the reproductive period (Figure 8).

## 4 Discussion

The external anatomy of the male reproductive tract in *L. podicipinus* is similar to patterns already described for the species (Bhaduri 1953). The testes are paired, whitish, and located ventrally to the kidneys. They are lined by the tunica albuginea and supported by another fibrous tunic, the mesorchium. Between the testes, EV can be seen leaving the testes in the direction of the kidneys.

### 4.1 Patterns and Variations

The internal morphology of the reproductive tract in *L. podicipinus* exhibits structural patterns similar to those observed in other species within the group (Hiragond and Saidapur 2000). In general, anurans show several different patterns in the organization of the male reproductive tract and the network of sperm transport channels. These patterns can range from channels with a joint urogenital function to single channels for genital and urinary functions (Blüm 1986). Among different species, structures, such as the seminiferous loculi and EV, are mostly similar, but there are also points of divergence, for example, the absence of ductules as the ID or LD, and even the presence of LCD (Hiragond and Saidapur 2000). These variations highlight the morphological variability present in the reproductive tract of anurans (Rheubert et al. 2017).

In the works of Blüm (1986), Oielska (2009), and Hiragond and Saidapur (2000), four patterns of structural organization of the male reproductive system in Anura were observed. Three of these patterns defined by Blüm (1986) are based on the Palearctic species of the genera *Bombina*, *Discoglossus*, and *Alytes*, where a developmental pattern of separation of the urinary and genital functions is indicated. In *Bombina*, the urogenital portion is connected through the canal system of both the genital and urinary portions; in *Discoglossus*, there is a loss of connection between testes and kidneys, and only the WD is shared; while in *Alytes*, there are different ducts that drain the organs (testes and kidneys) separately. Hiragond and Saidapur (2000) investigated six species from the Indo‐Malayan region, and the patterns found did not coincide with any of the three reported by Blüm (1986), since the eastern species have an intimate interaction between testes and kidneys, with the sperm being transported through the EV to the renal glomeruli, where they are taken to the WD. This connection was also found by Rheubert et al. (2017) in the male reproductive tract of the Nearctic species *A. catesbeiana*. The pattern observed in the Indo‐Malayan and Nearctic species appears to be closer to that found in the Neotropical species *L. podicipinus*.

### 4.2 Male Reproductive Tract in *L. Podicipinus*

In *L. podicipinus*, the seminiferous locules are drained by the ID, which emerge within the testicular stroma. These IDs have been observed in several species, such as *Euphlyctis cyanophlycts* (Dicroglossidae), *Fejervarya limnocharis* (Dicroglossidae) and *A. catesbeiana* (Ranidae) (Hiragond and Saidapur 2000; Rheubert et al. 2017). However, in other species, such as *Polypedates maculatus* (Rhacophoridae) and *Duttaphrynus scaber* (Bufonidae), IDs are absent, and the seminiferous locules instead empty directly into the EV network (Hiragond and Saidapur 2000).

In addition to the IDs, *L. podicipinus* possesses the LCD, a canal that extends longitudinally throughout the testis. This structure is similar to the one described for the species *P. maculatus* (Hiragond and Saidapur 2000) and is not present in the other species *E. cyanophlyctis*, *F. cyanophlyctis*, *F. limnocharis*, *A. catesbeiana* (Hiragond and Saidapur 2000; Rheubert et al. 2017). However, in *L. podicipinus*, the seminiferous locules do not radiate from the LCD as they do in *P. maculatus*. Instead, the IDs drain the locules and transport sperm until they converge in the LCD. The pattern observed in *P. maculatus* appears to resemble more closely the organization of the reproductive tract described for caecilian species (Wake 1968). In the species studied, the EV exits the testis and crosses the mesorchium toward the kidneys, in a similar way to that observed in other species (Hiragond and Saidapur 2000; Rheubert et al. 2017). Another characteristic observed *in L. podicipinus* and other species is the presence of sperm in the glomerulus. These traits appear to be well‐conserved, as all species with available histological descriptions in the literature exhibit these features. In the analysis of urogenital interaction in *L. podicipinus*, the EV network connects to the LDs, which are part of the reproductive kidney. Unlike what is observed in Ranidae and Dicroglossidae (Rheubert et al. 2017; Hiragond and Saidapur 2000), only a single lateral renal canal is present, where sperm are deposited before entering the renal corpuscles. However, the structure observed in *L. podicipinus* is similar to that found in other species, such as *P. maculatus* and *D. bengalensis* (Bufonidae) (Hiragond and Saidapur 2000). Histological analysis of the spermatic transport ducts in *L. podicipinus*, in addition to the description for *P. maculatus*, reveals that both species possess an LCD, a structure not reported in other anuran species described to date (Rheubert et al. 2017; Hiragond and Saidapur 2000).

### 4.3 Comparison Between Amphibian Species

In caecilians (Gymnophiona) and urodeles (Caudata), such as *C. thompsoni* and *A. maculatum*, the seminiferous lobules drain into a central LCD, which then branches into EV or sperm ducts that connect the testes to the kidneys (Figure 5). In contrast, anurans exhibit a different configuration, in which the IDs drain directly into the EV, with no LCD present. Interestingly, the branching of the central canal within the EV, a feature characteristic of both Caudata and Gymnophiona (Siegel et al. 2014; Serrano‐Pérez and Ramírez‐Pinilla 2021), is also observed in *L. podicipinus* and *P. maculatus*, but not in other anurans such as *A. catesbeiana*. Considering these findings, *L. podicipinus* shows morphological traits shared with apodes and urodele species (Figure 5). This contrasts with findings in other anuran species, where neither an LCD nor a central canal is present before the spermatic duct exits the testis. However, additional information on different species is needed to clarify how much variation exists among amphibians.

*Polypedates maculatus* and *L. podicipinus* species share some similarities in their reproductive behaviors. Both are foam‐nest breeders, and both the Rhacophoridae and Leptodactylidae families exhibit species with large testes and engagement in sperm competition (Kanamadi and Jirankali 1992; Girish and Saidapur 1999; Prado and Haddad 2003). While foam‐nest breeding may not be directly linked to multimale spawning (Prado and Haddad 2003), the similarities in reproductive conditions and behaviors may be associated with comparable morphological and physiological traits related to testicular sperm transport. It is known that reproductive characteristics can influence the morphology and physiology of anuran species (Furness et al. 2010; Byrne et al. 2015; Chen et al. 2023).

### 4.4 Seasonality and PCNA

Seasonal differences among individuals from the different periods analyzed were observed in the dilation of the IDs and in the amount of sperm present within them. In *S. acuminatus* (Hylidae), the reproductive structures of the kidneys showed anatomical changes during periods of higher reproductive activity (Olea et al. 2021). This finding may suggest a seasonal influence on the reproductive cycle of the species, similar to that observed in the IDs of the species studied. However, in *L. podicipinus*, the canals associated with the kidneys showed no anatomical changes between the periods analyzed, except for the variation in the amount of sperm in the lumen. The scarcity of information on the anatomy of the male reproductive tract in neotropical species during different periods of the reproductive cycle makes it difficult to obtain clear patterns for comparison.

Immunohistochemical analysis of proliferative cell activity was assessed by detecting positive cells for Proliferating Cell Nuclear Antigen (PCNA); PCNA‐positive cells indicate active cell proliferation. These cells were detected in individuals of the studied species throughout the year, regardless of the season, suggesting continuous proliferative activity. This result is similar to that observed in *Pelophylax esculentus* (Ranidae), *A. catesbeiana*, and *S. acuminatus* (Chieffi et al. 2000; Caneguim et al. 2013; Olea et al. 2021). In *P. esculentus*, spermatogonia and spermatocytes I were positive for PCNA labeling; in *L. podicipinus*, all early germ cells showed positive labeling. The prolonged labeling of PCNA, as well as its presence in the cytoplasm beyond the nucleus, may be related to its role in DNA repair reactions and interactions with other cytoplasmic proteins not yet identified, possibly kinases (Chieffi et al. 2000).

In *S. acuminatus*, PCNA‐positive labeling remained constant throughout the reproductive cycle. In contrast, *L. podicipinus* exhibited significant variation in PCNA labeling intensity, suggesting fluctuations in germinative cell activity. This occurred despite the species maintaining continuous testicular activity, as evidenced by the persistent presence of spermatozoa in the lumen across the reproductive cycle (Bordin et al. 2024b). Such fluctuations in germinative activity were also observed in *P. esculentus*, where cell proliferation increased during the summer and fall, periods of higher reproductive activity, unlike in *L. podicipinus* (Chieffi et al. 2000; Chianese et al. 2015).

In *A. catesbeiana*, there was an increase in PCNA labeling in spermatogonia in spring‐summer, associated with an increase in testosterone levels, which inhibit cell proliferation (Caneguim et al. 2013). This variation may be attributed to interspecific differences, methodological approaches used for immunolabeling quantification, and the origin of the individuals, which in *A. catesbeiana* were obtained from captivity (Caneguim et al. 2013), while those of *L. podicipinus* were collected from the wild. Although it is interesting to note, that while both species have continuous testicular activity, *A. catesbeiana* shows an increase in testicular size during the reproductive period (Sasso‐Cerri et al. 2004), which may influence the proliferation of spermatogonia due to seasonal testosterone levels. In contrast, *L. podicipinus* shows no variation in testicular size throughout the reproductive cycle, possibly due to its opportunistic reproductive behavior (Bordin et al. 2024b).

### 4.5 State of Reproductive Morphology in Anura

As discussed, genital morphology and urogenital connections exhibit considerable variation among anuran species. The scarcity of detailed information and the limited focus on these structures have hindered full exploration of their comparative potential and the identification of underlying patterns that need to be elucidated. Currently, the information available on the urogenital structure of anurans is limited, covering around 64 species from 21 families (Bhaduri 1953; Oielska 2009; Rheubert et al. 2017). However, the available information lacks histological details.

## 5 Conclusion

This study provides novel insights into the reproductive morphology of *L. podicipinus*, with a focus on cell proliferation and histological details of urogenital interactions, thereby expanding current knowledge about the morphology of the species. The findings highlight the complexity and variability within the group, emphasizing both similarities and differences in comparison to data available for other amphibian species. The reproductive characteristics observed in *L. podicipinus* offer valuable contributions to the understanding of anuran reproductive biology, an ecologically important group increasingly threatened by climate change and anthropogenic activities.

## Author Contributions

**Rafael O. A. Bordin:** conceptualization, methodology, formal analysis, writing – original draft, writing – review and editing, validation. **Classius de Oliveira:** conceptualization, methodology, validation, writing – original draft, writing – review and editing, project administration, supervision. **Raquel F. Domeniconi:** project administration, supervision, writing – original draft, writing – review and editing, conceptualization.

## Conflicts of Interest

The authors declare no conflicts of interest.

## Data Availability

The data is contained within the study.

## References

[jmor70072-bib-0001] de Almeida Prado, C. P. , M. Uetanabaro , and F. S. Lopes . 2000. “Reproductive Strategies of *Leptodactylus chaquensis* and *L. Podicipinus* in the Pantanal, Brazil.” Journal of Herpetology 34, no. 1: 135. 10.2307/1565249.

[jmor70072-bib-0002] Bhaduri, J. L. (1953). “Study of the Urinogenital System of Salientia.” Proceedings of the Zoological Society of Benga 6, no. 1: 110.

[jmor70072-bib-0003] Blüm, V. 1986. Vertebrate Reproduction. Springer Berlin Heidelberg. 10.1007/978-3-642-71074-2.

[jmor70072-bib-0004] Bordin, R. O. , C. E. S. Fernandes , L. Franco‐Belussi , T. R. F. Leão , and M. Sanabria . 2022. “Sperm Morphology and Testicular Histology of the Polyandric Species *Leptodactylus podicipinus* (Anura: Leptodactylidae) From an Urban Environment.” Anatomical Record 305, no. 12: 3532–3542. 10.1002/ar.24928. 35365960

[jmor70072-bib-0005] Bordin, R. O. A. , C. de Oliveira , and R. F. Domeniconi . 2024a. “Germinative Dynamic, Seasonality and Polyandry: A Dive in Neotropical Point‐Belly Frog Reproduction.” Anatomical Record. 10.1002/AR.25627. 39739379

[jmor70072-bib-0006] Bordin, R. O. A. , C. Oliveira , and R. F. Domeniconi . 2024b. “Immunolocalization of Aquaporin 1, 2, and 9 in Anuran Testis of the Neotropical Pointedbelly Frog *Leptodactylus podicipinus* .” Current Issues in Molecular Biology 46, no. 9: 9958–9969. 10.3390/cimb46090594. 39329946 PMC11430573

[jmor70072-bib-0007] Byrne, P. G. , C. Dunne , A. J. Munn , and A. J. Silla . 2015. “Environmental Osmolality Influences Sperm Motility Activation in an Anuran Amphibian.” Journal of Evolutionary Biology 28, no. 3: 521–534. 10.1111/jeb.12584. 25586700

[jmor70072-bib-0009] Caneguim, B. H. , J. S. Luz , S. R. Valentini , P. S. Cerri , and E. Sasso‐Cerri . 2013. “Immunoexpression of Aromatase and Estrogen Receptors β in Stem Spermatogonia of Bullfrogs Indicates a Role of Estrogen in the Seasonal Spermatogonial Mitotic Activity.” General and Comparative Endocrinology 182: 65–72. 10.1016/j.ygcen.2012.12.002. 23247274

[jmor70072-bib-0010] Canene‐Adams, K. (2013). “Preparation of Formalin‐fixed Paraffin‐embedded Tissue for Immunohistochemistry.” In Methods in Enzymology, 225–233. Academic Press. 10.1016/B978-0-12-420067-8.00015-5. 24182927

[jmor70072-bib-0011] Chaves, M. F. , G. J. B. Moura , F. C. M. A. Tenório , et al. 2017. “Influence of Rainfall and Temperature on the Spermatogenesis of *Leptodactylus macrosternum* (Anura: Leptodactylidae).” Zoologia 34: 1–7. 10.3897/zoologia.34.e20782.

[jmor70072-bib-0012] Chen, S. , Y. Jiang , L. Jin , and W. Liao . 2023. “Testing the Role of Natural and Sexual Selection on Testes Size Asymmetry in Anurans.” Biology 12, no. 2: 151. 10.3390/biology12020151. 36829429 PMC9952133

[jmor70072-bib-0013] Chianese, R. , V. Ciaramella , S. Fasano , R. Pierantoni , and R. Meccariello . 2015. “Kisspeptin Drives Germ Cell Progression in the Anuran Amphibian *Pelophylax esculentus*: A Study Carried out in Ex Vivo Testes.” General and Comparative Endocrinology 211: 81–91. 10.1016/j.ygcen.2014.11.008. 25452028

[jmor70072-bib-0014] Chieffi, P. , R. Franco , D. Fulgione , and S. Staibano . 2000. “PCNA in the Testis of the Frog, *Rana esculenta*: A Molecular Marker of the Mitotic Testicular Epithelium Proliferation.” General and Comparative Endocrinology 119, no. 1: 11–16. 10.1006/gcen.2000.7500. 10882544

[jmor70072-bib-0038] Cope, E. D. 1862. “On Some New and Little Known American Anura.” Proceedings of the Academy of Natural Sciences of Philadelphia 14: 151–159.

[jmor70072-bib-0016] Franco, J. R. , E. Dal Pai , M. V. C. Calça , et al. 2023. “Atualização Da Normal Climatológica E Classificação Climática De Köppen Para O Município De Botucatu‐Sp.” IRRIGA 28, no. 1: 77–92. 10.15809/irriga.2023v28n1p77-92.

[jmor70072-bib-0017] Furness, A. I. , R. W. McDiarmid , W. R. Heyer , and G. R. Zug . 2010. “Oviduct Modifications in Foam‐Nesting Frogs, With Emphasis on the Genus Leptodactylus (Amphibia, Leptodactylidae).” South American Journal of Herpetology 5, no. 1: 13–29. 10.2994/057.005.0102.

[jmor70072-bib-0018] Girish, S. , and S. K. Saidapur . 1999. “Mating and Nesting Behaviour, and Early Development in the Tree Frog *Polypedates maculatus* .” Current Science 1, no. 76: 91–93.

[jmor70072-bib-0019] Gomes, A. D. , R. G. Moreira , C. A. Navas , M. M. Antoniazzi , and C. Jared . 2012. “Review of the Reproductive Biology of Caecilians (Amphibia, Gymnophiona).” South American Journal of Herpetology 7, no. 3: 191–202. 10.2994/057.007.0301.

[jmor70072-bib-0020] Hiragond, N. C. , and S. K. Saidapur . 2000. “The Excurrent Duct System of Sperm Transport in *Rana cyanophlyctis*, *Rana limnocharis*, *Polypedates maculatus*, *Microhyla rubra*, *Bufo melanostictus* and *Bufo fergusonii* .” Zoological Science 17, no. 4: 453–458. 10.2108/0289-0003(2000)17[453:TEDSOS]2.0.CO;2.

[jmor70072-bib-0021] Kanamadi, R. D. , and C. S. Jirankali . 1992. “Testicular Activity in *Polypedates maculatus* (Rhacophoridae): Seasonal Changes in Spermatogenesis and Fat Bodies.” Journal of Herpetology 26, no. 3: 329. 10.2307/1564890.

[jmor70072-bib-0023] Lombardi, J. 1998. Comparative Vertebrate Reproduction. Springer US.

[jmor70072-bib-0024] Méndez‐Tepepa, M. , C. Morales‐Cruz , E. García‐Nieto , and A. Anaya‐Hernández . 2023. “A Review of the Reproductive System In Anuran Amphibians.” Zoological Letters 9, no. 1: 3. 10.1186/s40851-023-00201-0. 36782341 PMC9926845

[jmor70072-bib-0025] Oielska, M. 2009. *Reproduction of Amphibians (1st edition)*. CRC press. 10.1201/9781482280135.

[jmor70072-bib-0026] Olea, G. , E. Cheij , A. P. C. Boccioni , F. Rodriguez , J. Céspedez , and D. Lombardo . 2021. “Gametogenesis and Reproductive Dynamics of *Scinax acuminatus* (Anura: Hylidae): Morphological, Histological and Immunohistochemical Analysis.” Anais da Academia Brasileira de Ciências 93, no. 2: 1–11. 10.1590/0001-3765202120190841. 34190842

[jmor70072-bib-0027] Prado, C. P. A. , and C. F. B. Haddad . 2003. “Testes Size in Leptodactylid Frogs and Occurrence of Multimale Spawning in the Genus *Leptodactylus* in Brazil.” Society 37, no. 2: 126–131. 10.1670/0022-1511(2003)037.

[jmor70072-bib-0028] Pucci Alcaide, A. , F. Pucci Alcaide , A. A. Michel , and M. L. Ponssa . 2020. “Testicular Histology of Anurans That Deposit Eggs out of Water.” Acta Zoológica Lilloana 64, no. 2: 84–115. 10.30550/j.azl/2020.64.2/2.

[jmor70072-bib-0029] Rheubert, J. L. , H. E. Cook , D. S. Siegel , and S. E. Trauth . 2017. “Histology of the Urogenital System in the American Bullfrog (*Rana catesbeiana*), With Emphasis on Male Reproductive Morphology.” Zoological Science 34, no. 5: 445–451. 10.2108/zs170060. 28990475

[jmor70072-bib-0030] Sá, R. O. D. , T. Grant , A. Camargo , W. R. Heyer , M. L. Ponssa , and E. Stanley . 2014. “Systematics of the Neotropical Genus *Leptodactylus* Fitzinger, 1826 (Anura: Leptodactylidae): Phylogeny, the Relevance of Non‐Molecular Evidence, and Species Accounts.” In South American Journal of Herpetology, S1–S28. Sociedade Brasileira de Herpetologia.

[jmor70072-bib-0031] Santos, L. R. S. , L. Franco‐Belussi , and C. Oliveira . 2011. “Germ Cell Dynamics During the Annual Reproductive Cycle of *Dendropsophus minutus* (Anura: Hylidae).” Zoological Science 28, no. 11: 840–844. 10.2108/zsj.28.840. 22035307

[jmor70072-bib-0032] Sasso‐Cerri, E. , F. P. de Faria , E. Freymüller , and S. M. Miraglia . 2004. “Testicular Morphological Changes During the Seasonal Reproductive Cycle in the Bullfrog *Rana catesbeiana* .” Journal of Experimental Zoology Part A: Comparative Experimental Biology 301, no. 3: 249–260. 10.1002/jez.a.20023. 14981784

[jmor70072-bib-0033] Serrano‐Perez, C. A. , and M. P. Ramírez‐Pinilla . 2021. “Morphology and Histology of the Male Reproductive Tract of *Caecilia thompsoni* (Amphibia: Gymnophiona).” Anatomical Record 304, no. 5: 1119–1135. 10.1002/ar.24527. 33022119

[jmor70072-bib-0034] Siegel, D. S. , R. D. Aldridge , J. L. Rheubert , K. M. Gribbins , D. M. Sever , and S. E. Trauth . 2013. “The Testicular Sperm Ducts and Genital Kidney of Male *Ambystoma maculatum* (Amphibia, Urodela, Ambystomatidae).” Journal of Morphology 274, no. 3: 344–360. 10.1002/jmor.20100. 23192852

[jmor70072-bib-0035] Siegel, D. S. , A. E. Nicholson , B. Rabe , B. Beran , and S. E. Trauth . 2014. “The Evolution of the Sperm Transport Complex in Male Plethodontid Salamanders (Amphibia, Urodela, Plethodontidae).” Copeia 2014, no. 3: 489–502. 10.1643/CG-14-026.

[jmor70072-bib-0036] Wake, M. H. 1968. “Evolutionary Morphology of the Caecilian Urogenital System. I. The Gonads and the Fat Bodies.” Journal of Morphology 126, no. 3: 291–331. 10.1002/jmor.1051260303. 5750667

[jmor70072-bib-0037] Weibel, E. R. 1963. “Principles and Methods for the Morphometric Study of the Lung and Other Organs.” Laboratory Investigation: A Journal of Technical Methods and Pathology 12: 131–155. http://ci.nii.ac.jp/naid/10030916018/en/. 13999512

